# A Micro-Configured Multiparticulate Reconstitutable Suspension Powder of Fixed Dose Rifampicin and Pyrazinamide: Optimal Fabrication and In Vitro Quality Evaluation

**DOI:** 10.3390/pharmaceutics15010064

**Published:** 2022-12-25

**Authors:** Penelope N. Rampedi, Modupe O. Ogunrombi, James Wesley-Smith, Oluwatoyin A. Adeleke

**Affiliations:** 1 Division of Pharmaceutical Sciences, School of Pharmacy, Sefako Makgatho Health Sciences University, Pretoria 0208, South Africa; 2 Department of Clinical Pharmacology and Therapeutics, School of Medicine, Sefako Makgatho Health Sciences University, Pretoria 0208, South Africa; 3 Electron Microscope Unit, Sefako Makgatho Health Sciences University, Pretoria 0208, South Africa; 4 Faculty of Health, College of Pharmacy, Dalhousie University, Halifax, NS B3H 4R2, Canada

**Keywords:** tuberculosis, paediatric, antitubercular, pharmaceutical formulations, dosage form, multiparticulate drug carrier, dry suspension, oral drug delivery, design of experiments

## Abstract

The scarcity of age-appropriate pharmaceutical formulations is one of the major challenges impeding successful management of tuberculosis (TB) prevalence in minors. To this end, we designed and assessed the quality of a multiparticulate reconstitutable suspension powder containing fixed dose rifampicin and pyrazinamide (150 mg/300 mg per 5 mL) which was prepared employing solid–liquid direct dispersion coupled with timed dehydration, and mechanical pulverization. The optimized formulation had a high production yield (96.000 ± 3.270%), displayed noteworthy powder flow quality (9.670 ± 1.150°), upon reconstitution the suspension flow property was non-Newtonian and was easily redispersible with gentle manual agitation (1.720 ± 0.011 strokes/second). Effective drug loading was attained for both pyrazinamide (97.230 ± 2.570%*w*/*w*) and rifampicin (97.610 ± 0.020%*w*/*w*) and drug release followed a zero-order kinetic model (*R*^2^ = 0.990) for both drugs. Microscopic examinations confirmed drug encapsulation efficiency and showed that the particulates were micro-dimensional in nature (n < 700.000 µm). The formulation was physicochemically stable with no chemically irreversible drug-excipient interactions based on the results of characterization experiments performed. Findings from organoleptic evaluations generated an overall rating of 4.000 ± 0.000 for its attractive appearance and colour 5.000 ± 0.000 confirming its excellent taste and extremely pleasant smell. Preliminary cytotoxicity studies showed a cell viability above 70.000% which indicates that the FDC formulation was biocompatible. The optimized formulation was environmentally stable either as a dry powder or reconstituted suspension. Accordingly, a stable and palatable FDC antimycobacterial reconstitutable oral suspension powder, intended for flexible dosing in children and adolescents, was optimally fabricated.

## 1. Introduction

One of the main threats to world health that causes morbidity and mortality is tuberculosis (TB). It ranks number 13th on global causes of death. It is the second leading cause of death from a single infectious agent after COVID-19, surpassing the Human Immunodeficiency Virus/Acquired Immunodeficiency Syndrome (HIV/AIDS) [1,2]. The bacillus, *Mycobacterium tuberculosis* (*Mtb*), which causes TB, can affect any area of the body, although the lungs are where it most frequently manifests itself. The highest incidence of TB is seen in emerging nations, where socioeconomic issues including increasing urbanization and migration present unique difficulties for TB prevention and control [3,4]. In 2021, more than 10.6 million people were estimated to be infected with *Mtb* worldwide. Globally, over 1.2 million children were reported to have been ill with TB in 2021 [2]. Besides, in the past year, worldwide, approximately 1.1 million deaths were officially classified to be due to TB infections in HIV-negative individuals and 16% of these were children under 15 years [2,4,5,6]. The widespread limited availability of effective drug products that are well suited for use in children and historical exclusion of minors from clinical efficacy trials remains a major reason for treatment complications and mortality recorded for this age group [1,5,7,8].

In certain facets of pharmacotherapy, children differ from adults in so many ways which include drug-administering skills, medicine-related toxicity, and taste preferences. Based on biological and metabolic changes that occur during growth, the International Conference on Harmonization (ICH) divides the paediatric population into subpopulations. These include new-born infants (0–27 days); infants and toddlers (28 days–23 months); school children (2–11 years: split into preschool ages 2–5 years and school children ages 6–11 years) and adolescents (12–19 years) [9]. Premature new-born babies are the most fragile and heterogeneous paediatric group due to major shifts in development in the last weeks of gestation, while full term new-born babies do not exhibit fully evolved metabolic machinery [10,11,12]. In addition, each subgroup shows variations in gastrointestinal pH, intestinal motility, bile salt conjugation and transport relative to adults; making the design of drug delivery systems complicated and the target population very restricted. Paediatric medications must be designed to better match a child’s age, size (height and mass), physiological state, treatment needs and taste/flavor preferences (i.e., organoleptic qualities). In addition, differences in administration routes, formulation category and drug dose may be needed to ensure appropriate treatment for all children [13,14,15]. These diverse requirements make it difficult to administer treatment within the different paediatric age groups, thus making it challenging to combat this infectious disease in children [9,14,15]. It is also more difficult to achieve the desired pharmacotherapeutic results for this age group due to other treatment-related difficulties like the prolonged duration and drug regimen complexity that can influence compliance and unwanted side effects mostly associated with drugs for resistant TB infection [5]. 

The WHO recommends the use of fixed-dose combinations (FDC) in the treatment of TB as the mixture of more than one drug in a combination formulation is known to improve compliance in multidrug therapy scheduled for extended periods [16,17]. In addition to simplifying the prescription process and distribution chain in the healthcare systems, fixed-dose combination preparations can aid the reduction of pill burden [18,19]. They have also been documented to reduce the risk of developing resistance related to the therapy of infectious diseases by preventing the use of single drug entities [20]. However, until the end of 2015, there were no antitubercular drug products designed according to WHO’s revised body weight dosing guidelines for first-line drugs in children. This paediatric treatment regimen is usually based on the combination of three active pharmaceutical ingredients (APIs) namely—isoniazid, pyrazinamide, and rifampicin while ethambutol is mostly excluded from the FDC formulations as its use is recommended to be with caution due to its potential to cause undesirable effects [21,22]. In 2015, WHO increased the daily doses for isoniazid, pyrazinamide and rifampicin to 10 mg/kg, 35 mg/kg, and 15 mg/kg, respectively because lower doses triggered increase in drug resistance and dose inefficiency? On this basis, the quantity of active drug dose per tablet would typically be 50 mg, 150 mg, and 75 mg of isoniazid, pyrazinamide, and rifampicin, respectively. Scientist however considered these total doses unsuitable for children of all ages and may lead to incidences of under or overdosing [22,23,24]. 

Moreover, treatment strategies to date usually make use of commercially available adult solid dosage forms (e.g., tablets), which may pose the risk of choking especially for infants and young children. Consequently, clinical practices globally usually administer these unsuitable preparations by dividing them into segments, crushing and mixing with food, milk, water, or other liquids or in some cases compounding them extemporaneously in pharmacies for easy use in paediatric patients. These actions may result in dose inaccuracies, instability of active drugs, reduced bioavailability, patient non-compliance and drug resistance [8,24,25,26]. Thus, developing innovative and child-friendly pharmaceutical formulations suitable for paediatric TB management remains a pressing global need. 

Reconstitutable suspensions are unique dosage forms in which poorly soluble drugs (or even hydrophilic drugs) and suitable auxiliary materials are formulated into a dry powder or granule form. This dry particulate formulation is then dispersed into a liquid preparation for oral administration by directly mixing it with potable water at the point of dispensing [27,28]. They are widely accepted for use in people of all age groups, particularly children of all age brackets, thus potentially capable of promoting compliance and better therapeutic outcomes. They have the advantages of solid dosage forms such as being convenient to carry, easy to transport from one location to another and good stability. Likewise, dry suspensions can be administered easily and are especially suitable for paediatric and geriatric patients who have difficulty in swallowing [29]. This pharmaceutical formulation also helps with addressing stability issues associated with other commercially available solutions and suspensions such as drug decomposition due to prolonged contact between drugs and excipients in an aqueous medium [30]. Furthermore, the delivery of drugs via the oral route has long been known as a safe and convenient administration site. Relative to the other channels of drug administration, the oral route has attracted much attention because of its specific merits that encompass prolonged and tunable delivery, easy application, practicality of applying solid formulations, and better patient adherence. Drug administration via the oral route is convenient, safe, affordable, and suitable for use in children as well [31,32,33].

A review of current literature reveals that no study centered on the design of a fixed dose combination multiparticulate reconstitutable suspension formulation or a commercialized variant of such, containing first line antitubercular agents, specifically the pyrazinamide and rifampicin covered herein, has been reported. This study therefore aims to develop, optimize, and perform in vitro systematic evaluation of a fixed-dose combination reconstitutable oral suspension containing two first line TB antibiotics, namely pyrazinamide and rifampicin, at levels that align with the current WHO dosing recommendations for paediatric patients. The FDC reconstitutable oral suspension was formulated using various component ratios of polymeric excipients, sweetener, monovalent salt combinations and food grade strawberry flavor employing a direct solute-solvent incorporation approach coupled with dehydration and dry milling. A fixed-dose of rifampicin and pyrazinamide specified at 150 mg/300 mg per 5 mL, respectively were added to a bi-phased, uniformly dispersed water-ethanol based slurry containing both polymeric and non-polymeric excipients. Initial formulation synthesis was founded on a one-variable-at-a-time approach that was then embedded into a robust 3-factor, 3-level, 3-centrepoint Box–Behnken experimental design template using the Minitab^®^21 Statistical Software (Minitab LLC, State College, PA, USA). Accordingly, fifteen reconstitutable oral suspension FDC formulations were developed utilizing select response parameters which included percentage yield (Y%), angle of repose (AR degrees), average resuspension rate over 4 days (ARR strokes/second) and sedimentation indicator (F); all determined under ambient conditions. Formulation optimization was achieved employing the response surface optimizer by applying constraints on the abovementioned four response parameters. Subsequently, the optimization process was validated by preparing and testing this prototype formulation in triplicate. Post validation process, the environmental stability of the optimized FDC formulation (dry and reconstituted) was investigated, its biocompatibility with representative cell lines assessed and lastly; physicochemical and physicomechanical characteristics, including organoleptic qualities and microscopic surface morphology were evaluated. 

## 2. Materials and Methods

### 2.1. Materials

Magnesium stearate, glycerol, methylcellulose, potassium chloride, xylitol, sodium chloride, Kolliphor^®^ P188 (Poloxamer), polyvinyl alcohol, Ethyl-3-methyl-3-phenylglycidate (food grade strawberry flavor), polyvinylpyrrolidone (MW 10,000), sodium phosphate buffer, Dulbecco’s modified Eagle’s medium (DMEM), fetal bovine serum (FBS), L-glutamine, non-essential amino acids, penicillin, streptomycin amphotericin B and neutral red (NR) were obtained from Sigma-Aldrich, (St Louis, MO, USA). Rifampicin and ethanol were purchased from DB Fine Chemicals Ltd., (Sandton, South Africa) and Primellose^®^ from DFE Pharma, (Goch, Germany). Pyrazinamide was acquired from Glentham Life Sciences Ltd. (Corsham, UK). Hepatocyte (HepG2—ATCC^®^ HB-8065) cell lines were obtained from the American Type Culture Collection (ATCC) (Manassas, VA, USA). All other chemicals and reagents utilized were of analytical grade and used as received. 

### 2.2. Preparation of the FDC Reconstitutable Oral Suspension Formulations

#### 2.2.1. Building the Box–Behnken Experimental Design Template

Reconstitutable oral suspension formulation development was founded on a one-variable-at-a-time approach [34] that was then embedded into a robust 3-factor, 3-level, 3-centrepoint Box–Behnken experimental design template, a response surface method [35], using the Minitab^®^21 Statistical Software (Minitab LLC, State College, PA, USA). Accordingly, 15 formulations containing various concentrations of polymeric and non-polymeric excipients and a fixed quantity of model drug (i.e., rifampicin and pyrazinamide) were prepared in triplicates. The selected independent variables were combinations of additives namely: (i) *Factor A* (*X*_1_) consists of glycerol (GLY), ethanol (ETH), water (WAT); (ii) *Factor B* (*X*_2_) containing Primellose^®^ (PRIM), polyvinyl alcohol (PVA), polyvinylpyrrolidone (PVP), methylcellulose (MC), xylitol (XY), poloxamer (PX) and magnesium stearate (MS); and (iii) *Factor C* (*X*_3_) made up of potassium chloride (KCl), sodium chloride (NaCl) and food grade strawberry flavor (STRW). Besides, 3-levels of these independent variables described as lower (−1), middle (0) and upper (+1) limits (Table 1) were chosen for the establishment of the experimental design template as presented in Table 2. The choice of independent variables was based on their ability to produce easily resuspendable, free flowing dry particulate suspension powder. The selected response parameters or dependent variables were physicochemical parameters central to the performance of the reconstitutable suspension and these included percentage yield (Y_1_), angle of repose (Y_2_), average resuspension rate (Y_3_) and sedimentation indicator (Y_4_). Model evaluation and significance levels were carried out by employing the analysis of variance (ANOVA) where *p*-values less than 0.05 meant statistical significance.

#### 2.2.2. Fabrication of the FDC Reconstitutable Suspension Utilizing Laboratory Methods

The FDC reconstitutable oral suspensions were prepared using a direct solute-solvent incorporation approach coupled with dehydration and dry milling. All components, both polymeric and non-polymeric excipients of varying ratios were weighed on a calibrated balance (Radwag, Model AS220 R2, Radom, Poland) and added to a bi-phased, uniformly dispersed water-ethanol combination mixed by a magnetic stirring machine (Model H3 760-HSE, Lasec, Cape Town, South Africa) set at 500 rpm and a temperature of 35 ± 1 °C. The model drugs namely rifampicin and pyrazinamide, at a dosage of 150 mg/300 mg per 5 mL, respectively, were added to the polymeric slurry and stirred until homogeneity was achieved. The mixture was then transferred to a disposable flat pan and dried in the oven (Labex, Labdesign Engineering Pty Ltd., Robertville, Roodepoort, South Africa) at 60 ± 0.5 °C. The dehydrated mass was dry milled into fine powder employing a laboratory porcelain mortar and pestle (United Scientific Supplies, Inc., Libertyville, IL, USA). Reconstitution was accomplished by adding distilled water to the dry suspension powder under continuous manual shaking until an evenly dispersed slurry (containing 150 mg rifampicin/300 mg pyrazinamide per 5 mL) was formed.

### 2.3. Measurement of Response Parameters 

#### 2.3.1. Formulation Yield

The yield was calculated by relating the actual weight of each dry reconstitutable suspension powder after laboratory synthesis to its theoretical weight, which was based on its active and inactive components expressed as percentages [26,36]. All weights were determined using an analytical balance (AS220.R2; RadwagWagi Electroniczone, Radwag, North Miami Beach, FL, USA) and the calculation of percentage yield was based on Equation (1).

(1) $$\%\;\;\mathrm{Yield}\;=\frac{\;\mathrm{Actual}\;\;\mathrm{Yield}}{\;\mathrm{Theoretical}\;\;\mathrm{Yield}\;}\times 100$$

#### 2.3.2. Angle of Repose (Flowability)

The angle of repose is an important parameter for measuring the degree of flowability of dry particulates such as powders, granules, etc. [37]. In determining the angle of repose of the dry suspension powder, a glass funnel was affixed to a sheet of graph paper which was horizontally positioned on a flat, solid surface. The suspension powder was then carefully transferred through the neck of the funnel into its body until a point when it formed a cone-like pile. Thereafter, the radius (r) of the base of the pile and its height (h) were measured using a desk ruler, and the angle of repose (θ) was calculated utilizing Equation (2) [37].

θ = tan^−1^ h/r (2)

#### 2.3.3. Measurement of the Degree of Redispersibility

This was quantified as the resuspension rate (strokes/seconds) at two different time points and the average reading computed to represent the degree of redispersibility. Briefly the length of time required to manually reconstitute the dry suspension powder (2 g) in potable water (10 mL) on the first day and then to redisperse the same suspension after undisturbed storage under ambient conditions after four days were recorded. Reconstitution and redispersion were considered complete at the timepoints when the suspensions formed homogenous mixtures without traces of any residues. Dispersion was accomplished by gentle manual mixing (up and down motion) and logged as number of strokes while the duration elapsed was recorded in seconds [36,38] using a jumbo display digital countdown/countup timer (TR 112, Integrated Display Technology Ltd., Kowloon, Hong Kong) at the end of the first and fourth days. This investigation was performed in triplicates.

#### 2.3.4. Determination of Sedimentation Volume (Sedimentation Indicator)

Fifteen dry suspension powder samples (2 g each) were separately reconstituted to a final volume of 10 mL in a graduated stoppered glass vial using potable water. The produced suspensions were thoroughly dispersed by manually tossing the containing vials up and down over 30 s and the initial height was recorded (V_0_). Subsequently, each vessel was allowed to settle (i.e., separation into two layers) by storing it away undisturbed in the dark under ambient conditions (24 ± 3 °C) over four days. Thereafter, the sediment volume (V_u_) for each formulation was then measured and documented. All experiments were performed in triplicates and sedimentation indicator (F) was calculated as a ratio utilizing Equation (3) [39,40].

(3) $$\;F=\frac{V_{u\;}}{V_{0}}$$

### 2.4. Optimization of the FDC Reconstitutable Dry Suspension Formulation

The key goal of building this full quadratic experimental design template, which related independent with dependent variables, was to develop an optimal FDC reconstitutable oral suspension formulation. The Box–Behnken design, together with the response surface methodology were used to determine optimal response parameter levels by setting limits for predicting the choice FDC suspension formulation using the Minitab^®^21 statistical software (Table 3). Response parameters considered for the constrained simultaneous optimization process included percentage yield (Y_1_), angle of repose (Y_2_), average resuspension rate (Y_3_) and sedimentation indicator (Y_4_). The model appropriateness was confirmed using a two-way analysis-of-variance (ANOVA) and multiple correlation coefficient (*R^2^*). The composite desirability function of the four simultaneously optimized responses with a value closest to one was also utilized as an important indicator of the robustness of the optimization process. Furthermore, to authenticate the accuracy of this experimental design template, empirically derived responses generated by the optimized suspension formulation were compared with predicted values.

### 2.5. In Vitro Evaluation of the Optimized FDC Oral Suspension Formulation

#### 2.5.1. Quantification of Yield, Flowability, Resuspension Rate and Sedimentation Indicator

The percentage yield (by weight), angle of repose (a measure of dry powder flowability), sedimentation indicator which measured the volumetric changes in sedimentation behaviour of the reconstituted suspension before and after undisturbed storage over time, and the transitions in the rate of resuspension as it relates to time were quantified employing experimental strategies detailed above. 

#### 2.5.2. Drug Loading Efficiency

Pyrazinamide and rifampicin loaded reconstitutable suspension formulations and their placebos (5.0 g each) were separately dissolved in 100 mL of pH 7.4 phosphate-buffered saline (PBS) contained in a glass beaker. The resulting aqueous mixture was placed on a digital hotplate magnetic stirrer (Model H3760-HSE; Lasec; Ndabeni, Cape Town, South Africa) set at 35 ± 0.5 °C and 500 rpm to ensure complete dissolution. The samples were then visually monitored until a completely clear, homogenous solution was formed. Subsequently, 1 mL each of the resultant solutions (drug-loaded and placebo) were appropriately diluted using PBS and passed through a 0.45 µm nylon syringe filter (Whatman^®^, GD/X syringe filters, Sigma Aldrich, St. Louis, MO, USA). Collected sample filtrates were then separately analyzed by measuring absorbance using an ultraviolet (UV) spectrophotometer (Shimadzu UV 1280, Shimadzu Scientific Instruments, Columbia, MD, USA) independently programmed at maximum wavelengths (λ_max_) of 270 nm and 334 nm for pyrazinamide and rifampicin, respectively [41]. The filtered placebo solution was employed as a blank measurement to exclude interferences from background absorbances associated with the presence of excipients. The final absorbance values for pyrazinamide were measured separately at its own specific λ_max_ and obtained values were fitted into a linear calibration equation (i.e., *y* = 0.031*x*, *R*^2^ = 0.966) to obtain the actual percentage pyrazinamide content of the formulation. Thereafter, rifampicin was treated similarly, and its resultant absorbance values were processed using a different linear equation (i.e., *y* = 0.024*x*, *R*^2^ = 0.997). All quantifications were performed using three replicate samples. 

#### 2.5.3. Dissolution Studies and Drug Release Kinetics

The in vitro dissolution experiment was carried out on three separate optimized drug-loaded formulations by modifying previously published methods [42,43,44,45,46]. Drug-loaded suspensions (15 mL) were contained in a cellulose membrane dialysis tubing with a molecular weight cut-off of 14,000 Da (D9402-100FT, Sigma Aldrich, St. Louis, MO, USA) and placed into lidded glass baths containing 500 mL PBS. The entire contrivance was fitted into a shaking water bath (Model 821308, Scientific Laboratory Equipment, Johannesburg, South Africa) set at 37 ± 1 °C and 100 rpm. For each sample tested, 5 mL of the dissolution medium was collected and replaced with an equal volume of freshly prepared, temperature equilibrated PBS solution at predetermined time intervals (i.e., 5 and 30 min; 1, 2, 4, 6, 8, 10 and 12 h). Aliquots drawn were then diluted and filtered using 0.45 µm Whatman^®^ nylon syringe filter (Sigma Aldrich, St. Louis, MO, USA) and analyzed using an ultraviolet spectrophotometer (Shimadzu Scientific Instruments, Columbia, MD, USA) at λ*_max_* = 334 nm for rifampicin and 270 nm for pyrazinamide to detect drug absorbance which was eventually translated into percentage drug release values employing a linear polynomial equation (*y* = 0.024*x*; *R*^2^ = 0.997) and (*y* = 0.031*x*; *R*^2^ = 0.966) for rifampicin and pyrazinamide, respectively. 

The generated drug release profiles were further analyzed using mathematical models employing the KinetDS. version 3.0 open-source software. Based on a combination of robust validation quantities, including the correlation coefficient (*R^2^*) that is closest to one and the lowest Akaike Information Criterion (AIC) numerical value, release profile analyses and model of best fit choices were made. The model with the smallest AIC value was regarded as the model of best fit to the drug release profile and representation of the possible release mechanisms [47]. The models employed were zero-, first-, second order as well as Higuchi, Korsmeyer–Peppas and Michelis-Menten.

### 2.6. Optimized Formulation Characterization

#### 2.6.1. Differential Scanning Calorimetry

The temperature dependent transitioning patterns of the placebo, drug-loaded formulation, excipients, pyrazinamide, and rifampicin were assessed and compared utilizing a differential scanning calorimeter (DSC, Q2000 DSC, TA Instruments, New Castle, DE, USA). About 5.5 mg of each sample was weighed and placed in a standardized aluminum pan that was sealed with lid and place in the DSC machine. An empty aluminum pan was included as a reference for measurement purposes. All samples were analysed in triplicate at a rate of 10 °C/minute, heating temperature range of −65 °C to 300 °C and within an inert nitrogen gas infused environment set at a flow rate of 25 mL/minute. All generated thermographs were recorded and analysed accordingly [48]. 

#### 2.6.2. Thermal Gravimetric Analysis

The model drugs, excipients, placebo and optimized reconstitutable suspension formulation were evaluated using a thermogravimetric analyzer (TGA Q500 V20.13 Build 39, TA Instruments, New Castle, DE, USA). Around 9 mg of each sample were placed in different platinum pans ramped between 25 °C and 400 °C at a heating rate of 5 °C/minute under a nitrogen and air saturated environment. The percentage weight loss during each heating cycle was recorded as an average of three readings [49].

#### 2.6.3. Structural Elucidation

A Fourier transform infrared (FTIR) spectrophotometer (Perkin Elmer Spectrum 100 Series, Beaconsfield, UK) equipped with the Spectrum V 6.2.0 software was used to capture the spectra of the model drugs, excipients, placebo, and the optimized formulation in the transmission mode. Changes in the chemical backbone structure of each sample was recorded as spectra at a vibrational frequency range of 550–4000 cm^−1^. Each spectrum was generated as an average of 32 scans to achieve an acceptable signal-to-noise ratio and spectra resolution was maintained at 16 cm^−1^ [49].

#### 2.6.4. X-ray Diffractometry

The changes in the crystallinity of the model drugs, excipients, and placebo relative to the reconstitutable suspension formulation were monitored on an X’Pert Pro Powder X-ray diffractometer (PANalytical, Westborough, MA, USA). Machine divergence slit was fixed at 0.38 mm, measurements were executed using a reflection-transmission spinner and operations carried out using 1.54 Cu K-alpha radiation, 45 kV generator voltage, 40 mA tube current, 2θ range of 1–90° and 0.013 scan step size [50]. 

#### 2.6.5. Rheological Behaviour 

The rheological characteristics of the reconstituted drug-loaded formulation and its placebo was evaluated under ambient conditions (25 °C) using an Anton Paar MCR 302 Modular Compact Rheometer (Montreal, QC, Canada). About 10 mL of each sample was poured into a vessel and spindle set at a rotation speed programmed from 25 to 200 rpm. The viscosity was determined and a flow curve which represented a linear relationship between viscosity (mPa·s) and shear rate (s^−1^) was plotted [51]. 

### 2.7. Microscopic Analyses 

#### 2.7.1. Particle Surface Topography and Shape

The surface morphology of pure drugs placebo and drug-loaded formulations were viewed using the Zeiss Supra 55 VP Scanning Electron Microscope (Zeiss, Germany) at a 2 kV accelerating voltage. Firstly, the samples were cut into small parts, mounted on aluminium stubs using double sided adhesive carbon tape and then sputter coated with approximately 15 nm chromium for 45 s under argon atmosphere, using a Quorum T150 ES coater (Quorum, East Sussex, UK) to obtain an electrically conductive surface before imaging. The stubs with the four samples were then imaged using SEM to view the surface topography and shapes of the sample particles. Photomicrographs for all the samples were taken at the same magnification (1000×) for comparison purposes [52].

#### 2.7.2. Particle Size and Distribution

Images of the drug-loaded formulation, placebo, pyrazinamide, and rifampicin samples were captured using a Zeiss Discovery V20 Stereomicroscope (Zeiss, Germany) equipped with an Axiocam 503 camera. Contrast was optimised by imaging the placebo and pure drug pyrazinamide on a dark background because they are whitish in appearance, while the drug-loaded and pure drug rifampicin were viewed using a white background because of their reddish colour. Image J software (Version 1.8.0, National Institutes of Health, Bethesda, MD, USA) was used to calibrate images, segment particles using thresholding, binary and Watershed pre-processing steps, before measuring Feret’s diameter in each treatment. Ten pyrazinamide and rifampicin micrographs were separately analysed, and the results were subjected to statistical analysis using SPSS^®^ version 27 (IBM Corp, Armonk, NY, USA) [53,54]. 

### 2.8. Quantification of Surface Area and Porosity Parameters

The specific surface area, particle dimensions and pore diameters of the optimized and placebo formulations were quantified using the Brunauer-Emmett-Teller (BET) analyzer (Micromeritics TRISTAR II 3020, Micromeritics, Norcross, GA, USA) under a nitrogen atmosphere (adsorption–desorption isotherms at 77.350 K) within a cold free space (48.044 cm^3^). Approximately 0.3 g of each test sample was degassed under vacuum over 16 h at 40 °C [55]. 

### 2.9. Organoleptic Evaluations

The objective of this study was to evaluate the palatability of the reconstituted suspension based on taste, colour, smell/odour, and appearance. All these properties were evaluated by a 5-member panel of human volunteers. For taste assessment, all volunteers were requested to dip the tip of their tongue in the suspension for 15 s and then spit out. All panelists were provided with potable water to thoroughly rinse their mouths to remove any formulation residue before evaluating another sample (3 samples per panelist). The quality of the taste was evaluated using a 5-point scale with 1 = distasteful, 2 = slightly tasty, 3 = tasty, 4 = very tasty and 5 = excellent taste. For odour assessment, the volunteers were requested to briefly sniff the formulations without putting the formulation through their noses and the acceptability was evaluated using a 5-point hedonic scale with 1 = unpleasant, 2 = slightly pleasant, 3 = pleasant, 4 = very pleasant and 5 = extremely pleasant. The panel also assessed the appearance of the samples and rated them based on a 4-point scale of 1 = dissatisfactory, 2 = less satisfactory, 3 = satisfactory and 4 = very satisfactory. The colour of the formulation was evaluated by the same panel and graded using a 4-point scale of 1 = unattractive, 2 = slightly attractive, 3 = attractive and 4 = very attractive. An average numerical value indicating the overall acceptability of each evaluation of the formulations was computed [56,57]. Organoleptic evaluations of the dry suspension powder were also performed. The powder was evaluated for colour and smell, using the same 4-point scale for colour and 5-point scale for smell as mentioned above and the average numerical value indicating the overall acceptability of its physical presentation and colour was computed. 

### 2.10. Effect of Changes in Environmental Conditions on the Stability of the Dry and Reconstituted Suspension Powder

Environmental stability studies were performed on both optimized reconstituted and the dry powder suspension drug formulations over a period of 14 days and 8 weeks, respectively utilizing selected settings that were intended to simulate everyday use. Guidelines set by the International Conference on Harmonization (ICH) and WHO were factored into settings employed for this preliminary investigation. Both reconstituted and dry suspension samples were kept in different airtight, glass vials and stored as follows: (a) in a dark enclosure (25 °C ± 2 °C/60 ± 5% RH) (b) refrigerator (3 °C ± 2 °C) and (c) under room conditions (25 °C ± 2 °C/60 ± 5% RH) and assessed in triplicate. Rate of redispersibility, sedimentation, drug content, dissolution pH and organoleptic evaluations were selected as indicators for determining the influence of set storage conditions on the physical and chemical stability of these samples. All stability indicators were quantified at the end of 14 days and 8 weeks for the reconstituted suspension and dry powder, respectively and were compared to measurements conducted at the point when the sample were freshly prepared (i.e., day 0 where time = 0). Furthermore, at the end of 8 weeks, the powder suspension samples were reconstituted to evaluate their rate of redispersibility and dissolution pH [58]. 

### 2.11. In Vitro Cytobiocompatibility Evaluation of the Optimized Suspension Formulation

Cell viability testing was conducted on specimens of the drug-loaded formulation, placebo, pyrazinamide, and rifampicin using the hepatocyte cell lines (Hep G2—ATCC^®^ HB-8065^™^) and cancer coli-2 (Caco2 cells—ATCC^®^HTB-37^™^) as prototypes. Considering that the proposed administration route for the samples under investigation is oral, hence the respective selection of the liver, intestine, and kidney cell analogues for testing purposes. Researchers have reported the successful use of these cells as effective templates for the in vitro viability and toxicity effects of antitubercular and other bioactive agents [34,59,60,61,62]. The extent of cytotoxicity was quantified employing the Neutral Red (NR) cell viability assay [60]. The prototype cell lines were each seeded in a 96-well plate at a density of 40,000 cells/mL. The cells were kept overnight to aid attachment and thereafter exposed to test samples prepared at concentrations ranging between 0.0005 mg/mL and 50 mg/mL. After the 24 h of incubation with the test samples, the media was aspirated and then 20 µL of the neutral red solution was added to each well. The cultures were incubated for 3 h at 37 ± 0.5 °C in a humidified chamber saturated with 5% carbon dioxide). Post incubation, the cells were washed with pre-warmed PBS followed by 200 µL neutral red solution and left for 10 min under room conditions. Absorbance measurements were carried out at 540 nm using a micro plate reader (SpectraMax^®^ Paradigm^®^ Multi-mode Detection Platform, Molecular Devices, LLC, San Jose, CA, USA). The cytotoxicity of the test specimens was reported as a percentage viability in terms of the untreated control using a mathematical expression (%cell viability = [(sample − blank)/control − blank)] × 100). 

Data generated were expressed as means and standard deviations (SD). Normality of data was determined using the Kolmogorov–Smirnov test. Based on data normality, ANOVA test was used in the case of parametric datasets, and the Kruskal–Wallis ANOVA for non-parametric data to determine statistical significance. Statistical significance (*p* < 0.05) between test groups was evaluated using the Tukey and Dunn tests for parametric and non-parametric data, respectively. All statistical analyses were performed on GraphPad Prism (Version 5.0, San Diego, CA, USA).

## 3. Results and Discussion

### 3.1. Design of Experiments

The effective synthesis of 15 rifampicin-pyrazinamide loaded reconstitutable suspension formulations was facilitated by embedding the outcomes of the preliminary one-variable-at-a-time screening process into a more systematic 3-factor, 3-level, 3-centrepoints Box–Behnken experimental design template constructed utilizing the Minitab^®^ 21 statistical software. Using this robust strategy, we were able to identify the independent variables that had the most noticeable impact on the investigated response parameters (i.e., percentage yield, angle of repose, average resuspension rate and sedimentation indicator), thus limiting the number of experiments required to the barest minimum. Furthermore, the selection of the 3-centrepoints replicate sample minimized variations and errors within the design template and indicated the robustness of the design in evaluating quantified responses. 

Generally, the 15 dry suspension formulations were reddish brown powders that dispersed quite well in water during the reconstitution process. Their percentage yield ranged between 68.70% and 94.30% with the formulation that maximized sample recovery (highest yield) being the most desirable. Most samples had values >80% indicating the efficiency of the selected method of preparation and drug encapsulation. The angle of repose, a measure of powder flow or frictional force amongst loose powder particles, had values from 11.30° to 24.30° for all 15 formulations. According to pharmacopeial standards, low angle of repose corresponds to high flowability (i.e., free flowing powder) while the reverse is the case with higher values. All 15 formulations showed good flow properties as all recorded values were less than 30.00°. The sedimentation indicator was between 0.40 and 1.06 with the higher numerical value translating into a more stable, easily redispersible suspension with a reduced degree of sedimentation while lower values point to the opposite. Lastly, the average resuspension rate was 1.68–1.99 strokes/second meaning that generally, the formulations needed the application of minimal manual agitation over a short period to attain full homogeneity. The observed disparities in the magnitudes of the response parameters for each formulation can be associated with the noteworthy effects of the selected independent variables applied at different factor levels as it relates to the quadratic experimental design template employed. Table 4 details the values of the response parameters measured for all 15 formulations.

### 3.2. Process Optimization and Validation 

The independent variables were simultaneously optimized through the application of a set of constraints on the percentage yield, angle of repose, average resuspension rate and sedimentation indicator employing Minitab^®^ 21 statistical software. Model fitness and significance were assessed using the ANOVA approach in which case the responses were individually fitted into a full quadratic expression. The selected indicators included coefficients of determination (*R^2^*), *p*-values, desirability level for each response and compound desirability. Model fitting produced *R^2^* values close to 100%, *p*-values ≤ 0.05 (representing statistical significance of the impact of factor on responses) and a desirability level close to one signifying the robustness of the design template and its suitability as an optimization tool, particularly because of the complexity associated with processing multiple response parameters (Table 5). Through these actions, a formula, together with associated predicted responses, was generated for the preparation of the final optimized reconstitutable suspension (Table 5). Thereafter, the optimized dry suspension was prepared and tested in triplicate to further evaluate the accuracy of the design template by measuring the experimentally determined responses against the predicted values. Interestingly, both datasets showed a high degree of correlation thus corroborating the appropriateness of the selected optimization strategy.

### 3.3. In Vitro Assessment of the Optimized Suspension Quality and Release Behaviour 

#### 3.3.1. Quantification of Physical Parameters

The final optimized suspension formulation (RS_opt_) was a reddish-brown powder and had a high percentage yield of 96.000 ± 3.271%. RS_opt_ displayed good flowability typified by a low angle of repose of 9.670 ± 1.150°, as according to pharmacopeial standards, an angle of repose less that 30° depicts excellent powder flow properties. It’s degree of redispersibility was 1.720 ± 0.011 strokes/second and it had a sedimentation indicator of 1.00 ± 0.010, portraying that the suspension hardly underwent sedimentation upon storage and required minimal manual agitation over a short period to reach complete homogeneity. 

#### 3.3.2. Drug Content Uniformity

The optimized suspension contained 97.23 ± 2.57%*w*/*w* pyrazinamide and 97.61 ± 0.02%*w*/*w* rifampicin showing a high encapsulation efficiency and uniform distribution of drug molecules throughout the excipient matrix. These results indicate that the encapsulated drugs remained stable during and after preparation of the RS_opt_ and that negligible amount of these bioactive agents were lost during the formulation synthesis process. These outcomes suggest that the present method is suitable for the fabrication of the reconstitutable suspension formulation described here and that the fabricated suspension matrix encapsulated model drugs efficiently. 

#### 3.3.3. Dissolution Studies and Drug Release Kinetics

**Figure 1:**
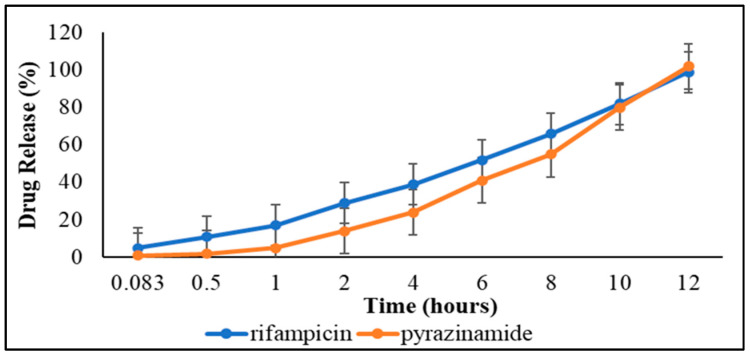
In vitro release of pyrazinamide and rifampicin represented in percentages over twelve hours under biorelevant conditions.

To understand the in vitro drug release behaviour, dissolution studies were carried out on the drug-loaded formulation containing an equivalence of 97.23 ± 2.57%*w*/*w* pyrazinamide and 97.61 ± 0.02%*w*/*w* rifampicin in pH 7.4 phosphate-buffered solution over 12 h under biorelevant conditions (Figure 1). Percentage cumulative drug release (% CDR) was calculated as the total amount of drug liberated from the optimized formulation matrix with an increase or decrease in % CDR representing a respective rise or decline in release rates, respectively. Within the first 5 min of dissolution, 5.00 ± 0.00% rifampicin and 1.00 ± 0.01% pyrazinamide was released from the amount encapsulated within the suspension formulation matrix. This represents the onset of matrix disintegration—a reflection that some pyrazinamide and rifampicin molecules adsorbed onto or incorporated near the particle surfaces. This would lead to a “burst effect” in vivo triggering the initiation of the desired pharmacological action that is then maintained by continuous drug release from the formulation over time [63]. The burst phase was then followed by a consistent increase in the release rate of both model drugs over time with total drug release (100%) accomplished at 12 h. 

As mentioned earlier, the selection of the best fitting model and representation of the possible release mechanisms was based on the fitted profile with the smallest AIC and highest *R^2^* (closest to 1) values (Table 6). On this basis, the zero-order kinetic model provided the best fit parameters for pyrazinamide (*R*^2^ = 0.99 and *AIC =* 48.77) and rifampicin (*R*^2^ = 0.99 and *AIC* = 40.75). This indicates that drug release from the formulation was consistent over time, irrespective of the initial drug concentration. The slowest release rate of these drugs could also be attributed to the strong intermolecular attraction of the polymer-based matrix, which then prevents the release of entrapped drug molecules. Therefore, the polymer matrix undergoes relaxation (disentangling polymer chains or disintegration) and release the trapped drugs (matrix dissolution) slowly and consistently over an extended period [64]. The zero-order drug release mechanism is useful in principle for maintaining constant drug levels in biological fluids and plasma, lowering the frequency of dose, enhancing patient compliance, and promoting the desired pharmacotherapeutic efficacy [47]. 

### 3.4. Physicochemical Characterization

#### 3.4.1. Determination of Thermal Stability

##### Differential Scanning Calorimetric Analysis

**Figure 2:**
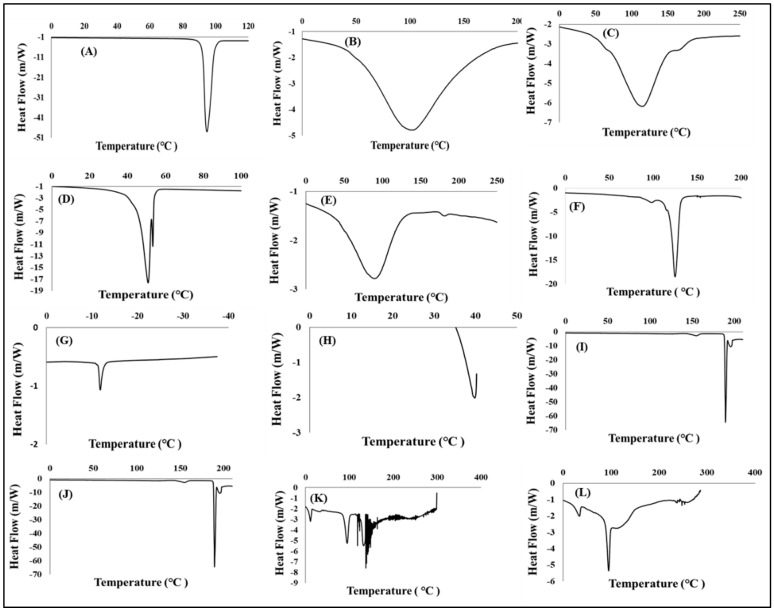
DSC thermographs of: (**A**) xylitol, (**B**) Primellose^®^, (**C**) polyvinylpyrrolidone, (**D**) methylcellulose, (**E**) Kolliphor^®^, (**F**) magnesium stearate, (**G**) potassium chloride, (**H**) sodium chloride, (**I**) pyrazinamide, (**J**) rifampicin, (**K**) placebo and (**L**) the drug-loaded formulation.

Generated differential scanning calorimetry (DSC) thermographs were employed in the assessment of thermal behaviour of model drugs, additives, the placebo, and drug loaded formulation. Typical DSC thermographs are shown in Figure 2A–L. The DSC scans of xylitol in Figure 2A displayed a sharp endothermic peak corresponding to its melting point (T_m_) at 94.79 °C whilst Primellose^®^ (Figure 2B) showed an overly broad endothermic peak corresponding to the T_m_ at 101.97 °C [65,66]. Following the endothermic trend is polyvinylpyrrolidone with a corresponding broad melting peak at 121.43 °C (Figure 2C) [67] Methylcellulose thermograph (Figure 2D) depicts multiple broad endothermic peaks, a prominent endothermic peak at 88.93 °C and a small endothermic peak at 179.95 °C. The presence of the two endothermic peaks indicate that methylcellulose is a combination of two differently natured polymers [68]. Kolliphor^®^ (Figure 2E) displayed an endothermic peak associated with T_m_ at 50.87 °C [69]. The thermograph in Figure 2F exhibits magnesium stearate in two endothermic events—one small peak at 99 °C, which may be attributed to water loss (dehydration process) and a sharper endothermic peak at 123 °C attributed to the melting of the compound [70]. Figure 2G,H represent thermal events recorded for potassium chloride and sodium chloride which exhibited endothermic peaks at −11.79 °C and 40 °C, respectively. Although the shape of the endothermic peaks seemed to agree with those found in the literature, the endothermic temperatures seemed to differ as the melting and cooling points appear in exceedingly elevated temperatures due to the differences in the parameters set up for those studies such as sample heating at high temperatures for extended periods [71]. Pure pyrazinamide (Figure 2I) showed two endothermic peaks at 156 °C attributed to solid–solid transitioning into its two crystal forms (polymorphs). Pyrazinamide transitions from β to α polymorph was evident in the second endothermic peak (T_m_) at 194 °C when the drug is in its α-polymorphic form [72,73]. Thermograph of rifampicin shown in Figure 2J displays an exothermic peak at 261.59 °C [74]. Thermal peaks identified for each excipient and pure drugs confirmed their purity and stability as individual entities before incorporation into the formulation mixture. 

The placebo in Figure 2K displays a mixture of broad and sharp endothermic peaks relating to its semi-crystalline nature that arises from the combination of the crystalline and amorphous additives mentioned above. Likewise, the drug loaded formulation in Figure 2L is semi-crystalline due to the combination and endothermic and exothermic peaks from the drugs and the excipients that represent a physical blend of crystalline and amorphous compounds occurring in their rightful melting temperatures. The peaks associated with the pure drugs, rifampicin, and pyrazinamide still retained their positions but at lower intensities and this may be because the drug molecules were completely solubilized in the formulation and the blending within the matrix with other excipients as these peaks overlapped. Regardless of this reduced intensity, the peaks remained stable and intact, and this further confirm that no new molecule is formed as the end-product. Accordingly, the drugs and the excipients seemed to display complete stability without any irreversible chemical interactions.

##### Temperature Dependent Gravimetry

**Figure 3:**
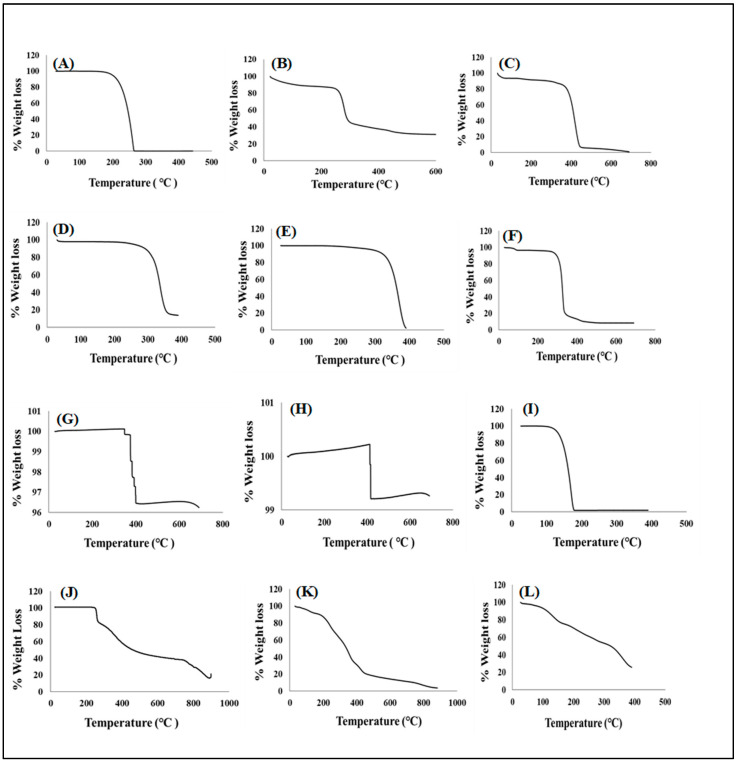
Thermogravimetric (TGA) curves of (**A**) xylitol, (**B**) Primellose^®^, (**C**) polyvinylpyrrolidone, (**D**) methylcellulose, (**E**) Kolliphor^®^, (**F**) magnesium stearate, (**G**) potassium chloride, (**H**) sodium chloride, (**I**) pyrazinamide, (**J**) rifampicin, (**K**) placebo and (**L**) the drug-loaded formulation.

Thermogravimetric curves of pharmaceutical excipients, pure drugs, optimized formulation, and placebo were recorded under nitrogen saturated atmosphere with purge, and heating rates of 40 mL/minute and 5 °C/minute, respectively are represented in Figure 3A–L. TGA analysis was conducted as an additional investigation of thermal degradation events measured as percentage weight loss as it relates to temperature changes with both serving as indicators of thermal stability for all test samples. First, crucial thermal events were identified for individual pharmaceutical excipients each model drug, additives, placebo, and drug loaded formulation. The initiation of xylitol thermal decomposition was observed at 200 °C and total weight loss recorded at 300 °C (Figure 3A) [75]. Both methylcellulose and Kolliphor^®^ showed their final thermal decomposition at temperatures greater than 400 °C with an onset of thermal events only displayed at higher heat levels (i.e., 300 °C and 330 °C, respectively) (Figure 3D,E)) [76,77]. The onset of thermal decomposition for Primellose^®^, polyvinylpyrrolidone, magnesium stearate, potassium chloride and sodium chloride were noted at 258 °C, 360 °C, 87 °C, 340 °C, and 400 °C, respectively while their final decomposition were recorded at temperatures of 600 °C and above (Figure 3B,C,F–H) showing their high resistance to thermal deformation as separate entities. Generally, the excipients showed relatively high single decomposition temperatures of 300 °C and above representing their own distinct stability and purity [78,79]. Pyrazinamide’s thermal breakdown was initiated at 125 °C and reached a point of complete thermal decay at 200 °C Figure 3I [80] while rifampicin showed more resistance to heat deformation with a thermal event starting at 240 °C and terminal weight loss continuing even at over 800 °C (Figure 3J) [74]. Thermal decomposition of the placebo and drug loaded formulation began at 200 °C and 125 °C, respectively with complete decay noted at ≥800 °C for placebo and ≥400 °C for the drug containing delivery system (Figure 3K,L). 

Interestingly, the placebo formulation displayed more heat resistance compared to the formulation matrix which contained the two non-polymeric, crystalline, and hydrophilic drug molecules (i.e., rifampicin and isoniazid). The observed pattern could be because of the drug molecules’ interaction with the blended polymeric chains embedded in the placebo in which the formulation matrix becomes more susceptible to chain disentangling and weight loss associated with temperature triggered depolymerization, chain scission and side group elimination resulting complete matrix decay at lower heating levels.

However, the drug free placebo is devoid of these interferences and majorly composed of a blend of multiple polymeric excipients which tend remain intact (minimal weight loss) for longer periods under higher heating effects/magnitudes [81,82]. This noticeable effect of the encapsulated drugs within excipient blended network (i.e., drug formulation) further showed that these molecules retained their activity and remained stable/intact within the formulation matrix. Overall, the TGA thermographs displayed the stability of the excipients as individual entities and as a blend (with the drugs) without any irreversible chemical transitions as salient thermal events occurred within ranges encompassing those of individual components.

#### 3.4.2. Infrared Absorption Spectroscopy

**Figure 4:**
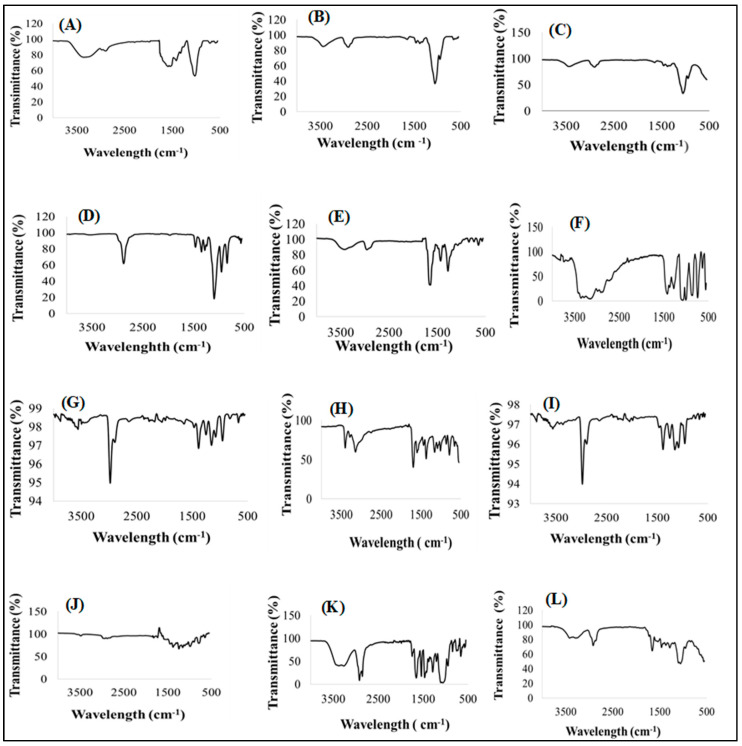
Fourier transform infrared spectroscopy of (**A**) Primellose^®^, (**B**) methylcellulose (**C**) magnesium stearate, (**D**) Kolliphor^®^, (**E**) polyvinylpyrrolidone, (**F**) xylitol (**G**) sodium chloride, (**H**) potassium chloride, (**I**) pyrazinamide, (**J**) rifampicin, (**K**) placebo and (**L**) the drug-loaded formulation.

To evaluate any drug-excipient interactions, Fourier transform infrared spectroscopy (FTIR) was performed on additives, both model drugs, drug loaded formulation and placebo and their spectra are shown in Figure 4A–L. The goal of the investigation was to find vibrational frequencies that indicated the presence of specific identifying functional groups in these samples using their respective spectra. Primellose^®^ indicated peaks related to O-H stretches at 3420 cm^−1^, C=O stretches at a vibrational frequency of 1582 cm^−1^ and C-O bends at 1062 cm^−1^ (Figure 4A) and this aligns with findings documented for Primellose^®^ by [83]. Specific peaks documented from methylcellulose show vibrations of 3490 cm^−1^ for O-H stretches, weak signal of C-H stretches at vibrations of 2864 cm^−1^ and C-O bends at 1060 cm^−1^ in Figure 4B [84]. Off note are peaks representing C=O weak stretches at 1576 cm^−1^, strong C-O bends at 1040 cm^−1^, C-H (CH_3_) stretches at 2954 cm^−1^ and O-H stretching at 3452 cm^−1^ from magnesium stearate in Figure 4C [84,85]. Subsequently, C-O strong bends at 1100 cm^−1^, O-H stretches at 2872 cm^−1^ and C-H stretches at 1354 cm^−1^ were observed from Kolliphor^®^ [86] in Figure 4D. Specific peaks were noted at 1658 cm^−1^ for C=O groups, weak C-H stretches at 2974 cm^−1^, C-N bends at 1282 cm^−1^ and 1418 cm^−1^ and O-H stretches at 3452 cm^−1^ for polyvinylpyrrolidone shown in Figure 4E [87]. Furthermore, specific peaks of very large stretching O-H groups were noted at vibrations of 3244–3364 cm^−1^, C-H stretches at 2924 cm^−1^ and C-C bends at 1432 cm^−1^ xylitol Figure 4F [88]. These peaks indicate asymmetric stretching bands of CH_3_ and CH_2_, respectively at vibrations of 2988 cm^−1^ and 2930 cm^−1^. Sodium and potassium chloride salts demonstrated identical peaks at similar vibrational frequencies with bonds representing stretching bands at vibrations of 1096 cm^−1^ [89,90,91] as illustrated in Figure 4G,H, respectively.

The pure drug pyrazinamide (Figure 4I) displayed specific peaks of N-H stretches at vibrations of 3414 cm^−1^ and 3154 cm^−1^, C=O stretches at 1698 cm^−1^, C=N bends at 1596 cm^−1^, C=C bends at 1374 cm^−1^ and C-N ring bends at 1166 cm^−1^ [34,80,92]. The pure drug rifampicin documented peaks at 3496 cm^−1^ representing N-H stretch, O-H stretches at 2984 cm^−1^, C=O (keto group) stretches at 1694 cm^−1^, C=O (amine group) at 1794 cm^−1^ and C=C ring stretches at 1456 cm^−1^ in Figure 4J [93,94,95]. The placebo FTIR spectra (Figure 4K) showed O-H stretches at 3420 cm^−1^ which could depict the presence of xylitol, Kolliphor^®^, and methylcellulose, Primellose^®^ and magnesium stearate. There are strong CH_3_ and CH_2_ stretches at 2928 cm^−1^ and 2930 cm^−1^, respectively indicating the presence of polyvinylpyrrolidone, xylitol, Kolliphor^®^, magnesium stearate, methylcellulose, sodium, and potassium chloride chemical backbone structure. Furthermore, peaks of C=O stretches at vibrations of 1658 cm^−1^ depict the presence of polyvinylpyrrolidone, magnesium stearate and Primellose^®^ whereas C=C and C-C stretches at 1544 cm^−1^ indicate the presence of xylitol. Peaks representing C-O stretches at vibrations of 1116 cm^−1^ confirms the presence of Primellose^®^, Kolliphor^®^P188, magnesium stearate and methylcellulose. The presence of C-N stretches appears at 1282 cm^−1^ and 1418 cm^−1^ showing the presence at polyvinylpyrrolidone. The last visible peaks appear as C-O-C oxirane rings at vibrations of 950 cm^−1^ for sodium chloride and potassium chloride. The given evidence showed that the placebo is a homogenous physical blend of the different excipients which may appear with slight shifts in the vibrational frequencies due to overlapping peaks from the same functional groups. 

**Figure 5:**
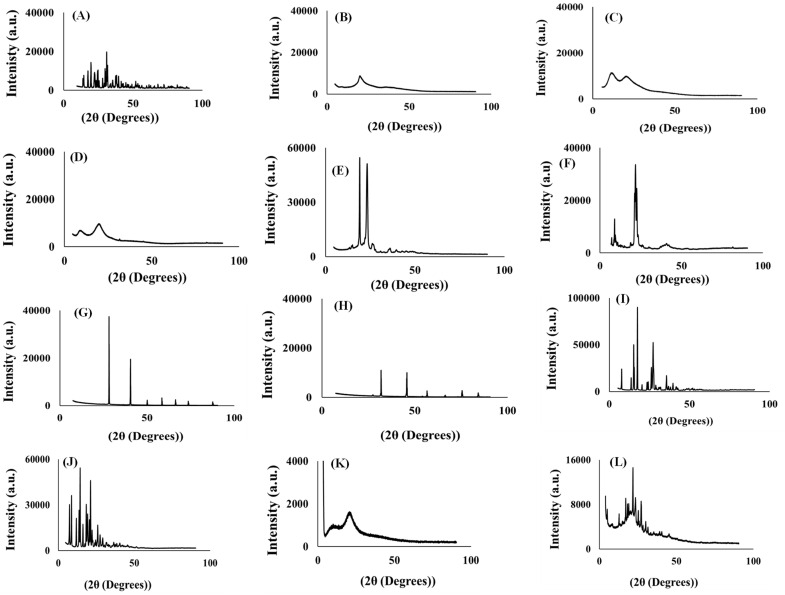
X-ray diffraction of (**A**) xylitol, (**B**) Primellose^®^, (**C**) polyvinylpyrrolidone, (**D**) methylcellulose, (**E**) Kolliphor^®^, (**F**) magnesium stearate, (**G**) potassium chloride, (**H**) sodium chloride, (**I**) pyrazinamide, (**J**) rifampicin, (**K**) placebo and (**L**) the drug-loaded formulation.

The FTIR spectra generated by the optimized drug loaded formulation (Figure 4L) showed peaks that represent the presence of functional groups characteristic of both pyrazinamide, and rifampicin located at different vibrational frequencies. Peaks such as N-H stretches appear at vibrations of 3246 cm^−1^ and 3340 cm^−1^ for both pyrazinamide and rifampicin, CH_3_ stretches at 2922 cm^−1^ and CH_2_ stretches at 2886 cm^−1^, C=O (amine group) stretches appear at 1674 cm^−1^, C=N ring stretches at 1454 cm^−1^, C-O stretches at 1112 cm^−1^, C-C stretches at 1464 cm^−1^ and C-O rings at 968 cm^−1^. The optimized drug loaded formulation spectra displayed distinctive peaks, appearing within identical vibrational frequency ranges obtained for the model drugs but with slight shifts due to the presence of polymeric and non-polymeric additives. The spectra generated for the optimum formulation showed functional groups specific to the pure drugs and/or excipients, revealing the level of structural compatibility among these components. This means that, despite the produced pyrazinamide and rifampicin’s relative amorphization as supported by XRD outputs in Figure 5, the typifying structural peaks remained noticeably unmodified, confirming the absence of chemically disruptive interactions during the formulation development process. Overall, the FTIR analysis revealed that the drugs and excipients were well integrated, compatible, and stable, with no destructive intermolecular or intramolecular interactions. 

#### 3.4.3. Powder X-ray Crystallography

The changes in matrix crystallinity between the active drugs, placebo, drug loaded formulation and excipients were further confirmed using X-ray diffraction analysis (XRD) with recorded diffractograms displayed in Figure 5A–L. Diffractograms recorded for xylitol showed high intensity, well defined sharp peaks between 20 and 32 (2θ) with intensities as high as (˃19,774 a. u.) (Figure 5A) validating its crystalline nature [96]. On the contrary, Primellose^®^ presented a broad peak at 20 (2θ) with a maximum intensity (≥8088 a. u.) which can be related to its amorphous characteristic, largely contributing to its non-crystalline domains within the molecular structure as indicated in Figure 5B [97]. In consensus with the amorphous structural character was polyvinylpyrrolidone, represented with the diffractogram in Figure 5C. The diffractogram unveiled broad peaks at 11 and 21 (2θ) with intensity as low as (˂11,500 a. u.) and the evidence was in accord with the results of Hotaby et al., 2017 [79]. Additionally, diffractograms shown in Figure 5D for methylcellulose also indicate two broader peaks with less intensity that extended as far as 9700 (a. u.) at an angle of 20 (2θ) with the lowest peak appearing at an intensity of 8667 a. u. and an angle of 19 (2θ). This indicates that the methylcellulose structure is amorphous and could possibly be attributed to the lack of crystalline regions within the molecular arrangement of the polymer [98]. However, Kolliphor^®^ (Figure 5E) is less crystalline, with some broad, spaced peaks throughout the diffractogram and two sharp peaks at 19 and 23 (2θ) [99]. Figure 5F displays a diffractogram that indicates magnesium stearate as a less crystalline and shows broad peaks spread throughout the diffractogram [100]. Diffractograms of potassium and sodium chloride in Figure 5G,H, respectively exhibited similar scattered trends with slight variations. Potassium chloride showed sharper diffraction peaks (˃30,000 a.u.) compared to sodium chloride (˃15,000 a.u.) showing that the structural arrangement of both salts is crystalline [101,102]. Figure 5I displays a diffractogram of pyrazinamide as a crystalline molecule with distinct, highly intense peaks and the longest peaks ˃80,000 at 18 (2θ) [103]. The diffractogram of rifampicin (Figure 5J) revealed its crystalline nature demonstrating high diffraction peaks at multiple (2θ) regions and peaks extend as high as 40,000 a.u. This gives evidence that rifampicin is a highly ordered crystalline molecule [104]. 

Diffractograms generated for the optimized drug and placebo formulations are considered a sum of individual diffractions generated by the respective excipients and model drugs. When compared to its drug loaded counterpart, the placebo diffractogram in Figure 5(K) irregularly exhibited slightly broader, sparse, and low intensity peaks ˂ 2000 a.u. with a combination of blunt regions both associated with crystalline and amorphous states attributed to the different mixtures of crystalline, semi-crystalline and amorphous excipients that were largely polymeric in nature. However, the drug-loaded formulation (Figure 5L) exhibited sharper and more intense peaks between 0–30 (2θ) range (>3000) and a less intense, broader contours across the rest of the (2θ) scale. This clearly gives evidence of the presence of the two drugs; pyrazinamide and rifampicin as crystalline molecules and appearing in their correct (2θ) scale as this indicates their encapsulation and stability within the powder formulation matrix. The rest of the less intense regions indicate the presence of a mix of mostly moderately crystalline or non-crystalline additives. The intensity of the peaks of pyrazinamide in Figure 5I and rifampicin in Figure 5J were reduced due to the peak overlap from all formulation (Figure 5L). 

#### 3.4.4. Rheological Behaviour 

**Figure 6:**
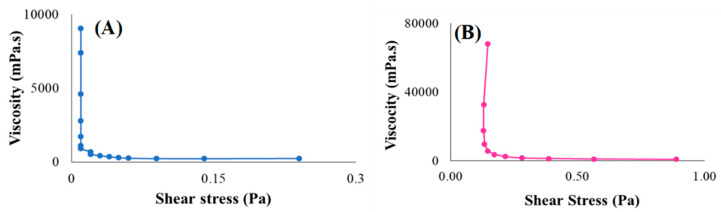
Rheological behaviour presented as viscosity versus shear stress for (**A**) placebo and (**B**) drug-loaded formulation.

The rheological characteristics of the reconstituted placebo and drug-loaded formulation was displayed as viscosity curves in Figure 6A,B, respectively. None of the flow curves between the placebo and drug-loaded suspension exhibited a linear relationship between the viscosity and shear rate thus demonstrating a non-Newtonian behaviour. The suspension’s rheological response was shear rate dependent, and the viscosity decreased with increasing shear stress [105]. However, the distinction between the two samples is that the drug-loaded formulation had lower mean viscosity (1923.55 mPa·s) compared to the placebo (13,179.81 mPa·s). The observed difference could be associated with the presence of the model drug molecules (i.e., rifampicin and pyrazinamide) within the polymeric matrix resulting in a noteworthy flow-modifying drug-polymer interactions. These interactions may occur in the suspension as ionic exchanges, hydrogen-bonding, dipole–dipole interactions, or hydrophobic interactions which have some effect on the formulation flow characteristics [106]. For instance, the non-ionic, amorphous polymer, polyvinyl pyrrolidone can be completely adsorbed onto the drug-loaded particles as a hydrodynamic layer to prevent molecular aggregation and sedimentation resulting from the presence of steric hindrances amongst particles thus making the suspension easily redispersible and allow excellent mobility/flow [107]. This can also be aided by the presence of stabilizers such as methylcellulose, that lowers surface tension between the particles and the dispersed medium by increasing the kinetic energy allowing free flow of the suspension thus lowering viscosity. 

Furthermore, the presence of electrolytes (sodium chloride—Na^+^Cl^−^ and potassium chloride—K^+^Cl^−^) act as flocculating agents by reducing the electric barrier between the particles in the liquid medium, through ionic interactions with both model drugs as they might hydrolyse when solubilising in the medium (forming an ionic complex with the electrolytes) and forms a bridge between adjacent particles to link them together in a loosely arranged structure. As a result, the system is deflocculated upon reconstitution in water, based on electrophoretic mobility potential, and because of the high force of attraction between neighbouring particles, lowering viscosity [108].

### 3.5. Microscopic Analyses 

#### 3.5.1. Surface Topography and Shape of Dry Suspension Powder

**Figure 7:**
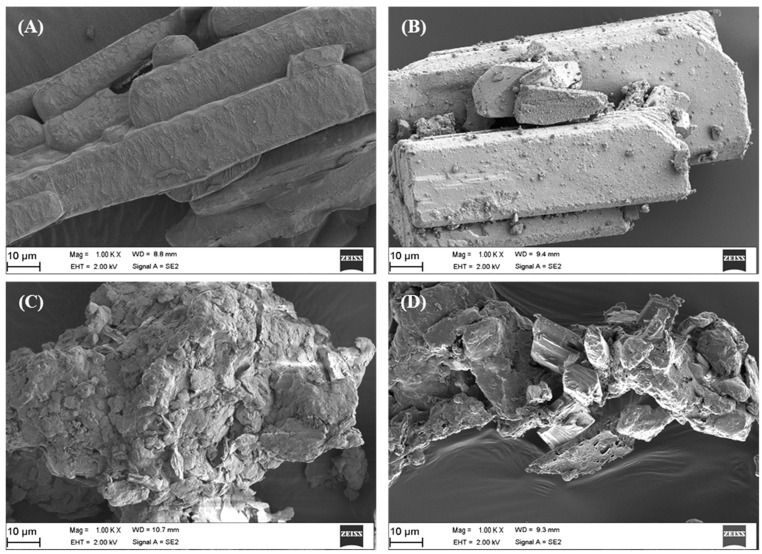
Scanning electron micrographs of: (**A**) pyrazinamide, (**B**) rifampicin, (**C**) placebo and (**D**) drug loaded suspension powder formulation captured at 1000×.

Scanning electron microscopy was used to assess the morphology of the drugs, placebo and that of the drug loaded suspension powder formulation (Figure 7A–D). The surfaces of the placebo particles were irregular, not well-demarcate and rough (Figure 7C) while its drug loaded counterpart displayed a matrix with intermittent protrusions of particulates that had distinguishing boundaries and an undulating topography. This may be attributed to the uniform incorporation/encapsulation of the drug molecules into the placebo matrix to form the drug loaded particulates (Figure 7D). 

#### 3.5.2. Microscopy Based Particle Size and Distribution Analysis

**Figure 8:**
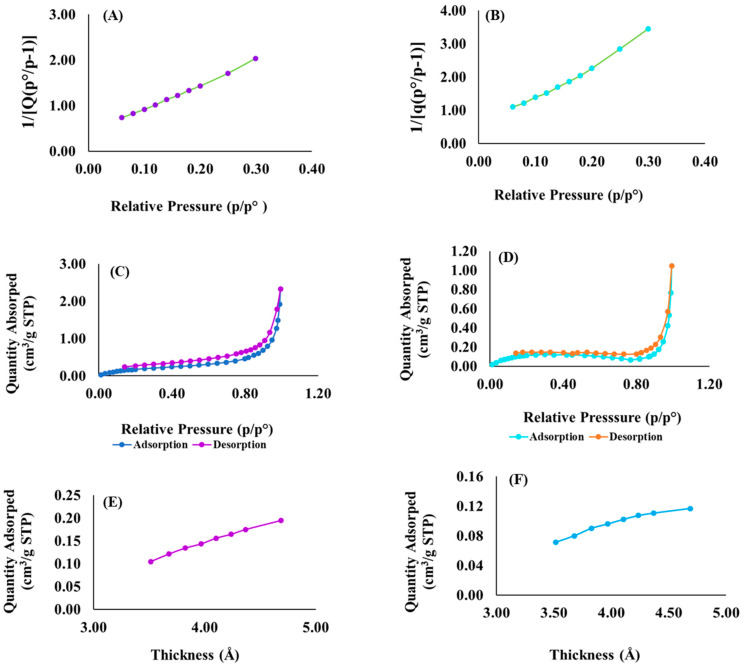
Surface area of (**A**) placebo and (**B**) drug-loaded formulation; isotherm curve displaying adsorption and desorption of (**C**) placebo and (**D**) drug-loaded formulation; thickness curve of (**E**) placebo and (**F**) drug-loaded formulation.

Stereomicroscopy and image analysis were used to assess the size distribution of particles in each of the treatments, and results are shown in Table 7. The Feret diameter of the drug-loaded suspension powder was higher than the placebo or drug particles, which further supports that the drugs rifampicin and pyrazinamide have been successfully fused and embedded within in the matrix of the placebo. These results were also in accordance with the BET surface area outputs in Figure 8, which showed that the surface area of the drug-loaded formulation was smaller and particle size larger than that of the placebo, all attributed to the successful encapsulation of the model drugs (Section 3.6). Furthermore, both analyses confirmed that both the placebo and its drug loaded counterpart were microparticulate in nature. 

### 3.6. Surface Area and Porosimetery Analyses

The surface areas of optimized drug loaded, and placebo formulations were obtained by employing the theory of Brunauer–Emmett–Teller (BET) on nitrogen adsorption isotherms generated from the sample surface measured at 77.35 K. The specific BET total surface area of the drug loaded formulation was 0.4284 m^2^/g while the placebo was 0.7559 m^2^/g and the single point surface area measured at 0.3780 m^2^/g and 0.6395 m^2^/g, respectively. The surface area of the drug-loaded formulation is smaller than the placebo as displayed in Figure 8A,B. This can be attributed to the successful encapsulation of pyrazinamide and rifampicin within the optimized formulation matrix. This is also supported by the recorded differences in their average sizes in which case the drug loaded formulation had larger particles measuring 140,039.78 Å (i.e., 14.00 µm) compared to the placebo that was 79,373.91 Å (i.e., 7.94 µm). The observed trend further validates the particle size estimation performed using the earlier described scanning electron microscopic evaluation which also showed that the drug loaded preparation had significantly larger mean particle size than the placebo. 

Furthermore, both the porosimeteric and microscopic analyses confirmed that both the placebo and its drug loaded counterpart were microstructured in nature. Besides, we also observed that as the numeric value of the particle size decreased, the surface area per unit volume increased. The adsorption–desorption studies revealed that the placebo had a larger average pore diameter (79.48 Å or 0.0079 µm) than the drug loaded (37.81 Å or 0.0038 µm) while they both exhibited the same thickness ranging between 3 nm and 5 nm. Thus, it can be inferred that rifampicin and pyrazinamide were embedded within the formulation matrix core and not adsorbed unto the outer shell of the particles. This possibly explains why the drug loaded formulation showed minimal burst effect followed by a consistent release pattern for pyrazinamide and rifampicin (Figure 1). Figure 8A–F are typical surface area, isotherm and thickness plots generated for samples tested.

### 3.7. Organoleptic Evaluations

As mentioned earlier, preliminary evaluations of organoleptic properties were based on taste, odour, appearance, and colour for the reconstituted suspension while the dry suspensions powder was only founded on colour and smell. The results indicated that on average the volunteers rated the suspension 5 ± 0 for taste and 5 ± 0 for odour, implying that the formulation had excellent taste and an extremely pleasant smell. Both the appearance and colour change were rated an average of 4 ± 0. This means that the appearance was extremely satisfactory and had an attractive colour. Furthermore, the panellists also rated the powder formulation in terms of its colour as 4 ± 0 indicating an attractive colour and a very pleasant smell given as 5 ± 0. Outcomes of this preliminary qualitative investigation makes the developed reconstitutable suspension and its dry powder form potentially attractive for paediatric use.

### 3.8. Environmental Stability of the Dry Powder and Reconstituted Suspension 

#### 3.8.1. Stability Evaluation of Dry Suspension Powder 

The evaluation of formulation stability was performed in triplicates per sample stored under varied environmental conditions: under room conditions (25 °C ± 2 °C/60 ± 5% RH); in a dark enclosure (25 °C ± 2 °C/60 ± 5% RH) and under refrigeration (3 °C ± 2 °C) over 8 weeks for the dry fixed dose suspension powder employing yield (%), angle of repose (degrees), drug content (%), colour change and odour as stability indicators. Results were reported as average ± standard deviation. At 8 weeks, samples separately stored in all three different storage conditions showed minimal variations in the stability indicators. The evaluation of the organoleptic properties (colour and odour) by volunteering panellists indicated a minimal change in the colour and odour from the samples stored under ambient conditions and refrigeration compared to that recorded at the start of the experiment (time = 0 weeks). The samples stored in an in the dark enclosure showed some discoloration and a slight decrease in its pleasant smell. Even though the stability indicators remained closely related, the slight colour change is undesirable considering patient compliance, making these conditions unsuitable for storage. Summarily, the suggested storage conditions for the dry powder would be in airtight vessels containing desiccant bags under ambient conditions or in the refrigerator (Table 8).

#### 3.8.2. Stability Testing of Reconstituted Suspension 

The stability of the reconstituted suspension was assessed under similar storage environments as the dry powder for 14 days mimicking storage duration for commonly used reconstituted antibiotic suspensions. The stability indicators considered here were rate of redispersibility, sedimentation, drug content (%) and pH. Furthermore, organoleptic properties namely taste, smell, appearance, and colour change were also evaluated during the test period. Stability testing was performed in triplicates and reported as average ± standard deviation. The stability indicators studied exhibited negligible changes in the numerical values for all storage conditions with slight variations in the pH (Table 9). According to the assessment made on the organoleptic properties, the most suitable storage conditions for the reconstituted suspension was the refrigerator or controlled ambient conditions (airtight and stored away from light sources). This then suggests that the dry suspension powder could be a potentially useful preparation for reconstitution purposes, especially for paediatric patients. 

### 3.9. Biocompatibility Assay on Select Cell Lines

**Figure 9:**
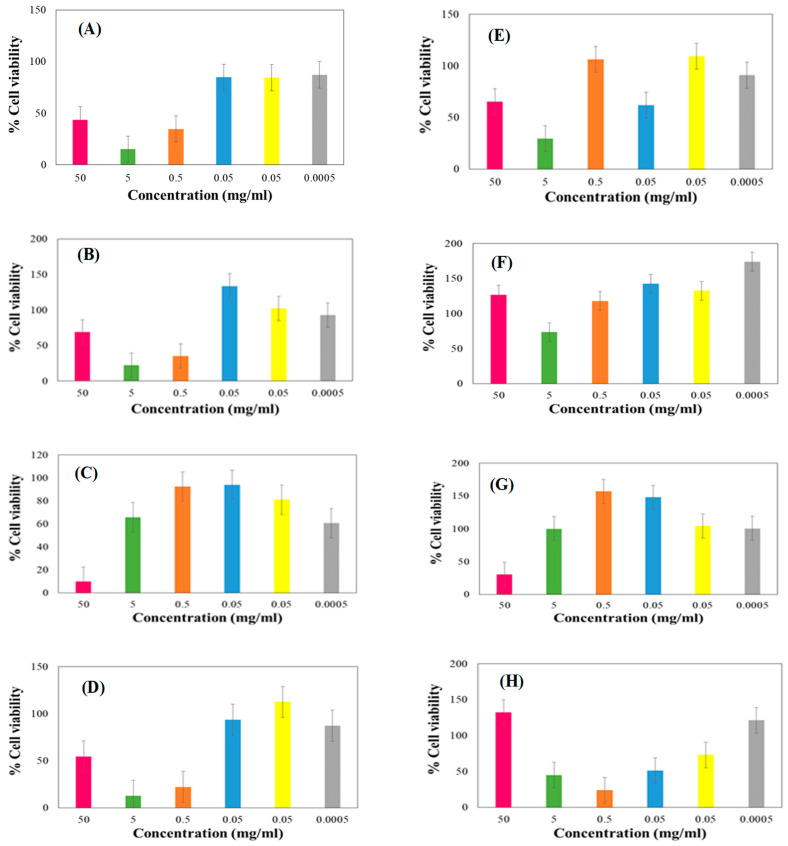
HepG2 cell biocompatibility levels measured by NR assay (**A**) placebo, (**B**) drug-loaded, (**C**) pyrazinamide and (**D**) rifampicin and Caco2 cell biocompatibility levels measured by NR assay (**E**) placebo, (**F**) drug-loaded, (**G**) pyrazinamide and (**H**) rifampicin.

Figure 9A–D illustrate the potential toxic effects of different concentrations of the test samples (i.e., the two active drugs, placebo and drug loaded formulation) on hepatocyte cells (HepG2) utilizing the neutral red (NR) assay method. A reduction in cell viability by more than 30% is considered a moderate cytotoxic effect while above the 70% point indicate good/desirable biocompatibility with the HepG2 cells. We noted that the cell viability decreased at higher concentrations of the placebo treatment (i.e., 5 and 50 mg/mL) and then subsequently increased (higher that 70%) at concentrations under 5 mg/mL (Figure 9A). A similar trend was noted for the drug loaded formulation solution at concentration above 5 mg/mL while HepG2 viability increased as sample concentration decreased. We observed a reduction in viability between concentrations of 0.05–0.0005 mg/mL but interestingly, obtained values were distinctly higher than 70% (Figure 9B). The cell viability increased with a decrease in pyrazinamide concentration up to 0.5 mg/mL while with lower concentrations, <0.5 mg/mL, cell viability decreased again but values were generally above the 70% mark indicated good compatibility. Besides, pyrazinamide solution with the highest concentration of 50 mg/mL had a very low cell viability (10.00 ± 1.40%) indicating that it triggered cell elimination at this elevated drug level. The second highest and the lowest concentration (5 and 0.0005 mg/mL) had cell viability values just underneath 70%. The other three concentrations (0.5, 0.05 and 0.005 mg/mL) yielded values that were all above 70%. For pyrazinamide there was a significant difference between cell viability values measured at 50 and 0.05 mg/mL. Generally, lower concentrations were considered to have no cytotoxic effect as their cell viability exceeded 70% with the neutral red assays. Finally, pyrazinamide showed no cytotoxic effect at concentrations 0.5 and 0.005 mg/mL (Figure 9C). Rifampicin, on the other hand, displayed an unusual pattern in which case cytotoxic effects were minimal at concentrations of 0.05 mg/mL and below with viability values above 70% but at higher concentrations above, 0.05 mg/mL (i.e., 0.5–50 mg/mL), a sharp increase in cytotoxic effects was observed with low viabilities, under 70% recorded (Figure 9D).

Figure 9E–H depict the impact of the different concentrations of the test sample solutions on the cancer coli-2 (Caco2) cell model. A fluctuating trend in the cell viability levels was observed for the placebo solution with a decrease in viability noted at 5 and 50 mg/mL and a rapid elevation observed with a further dilution of the placebo treatment to 0.5 mg/mL. Thereafter, a decrease was observed from 0.5 to 0.05 mg/mL and 0.005 to 0.0005 mg/ ml but all values at these four points were greater than 70% (Figure 9E). The cell viability decreased noticeably at the 5 mg/mL dilution of the drug loaded sample and then increased with a decrease in sample concentration but was unusually elevated at 50 mg/mL. Nevertheless, all values recorded at the different concentrations were still higher than 70% for the drug loaded formulation samples (Figure 9F). Pyrazinamide had a severe cytotoxic effect on the Caco2 cells at its highest concentration of 50 mg/mL while all concentrations less than 50 mg/mL were considered non-cytotoxic (Figure 9G). Rifampicin was generally toxic on the cells (viability < 70%) except for the lowest (0.0005 mg/mL), highest (50 mg/mL) and in-between (0.005 mg/mL) concentrations that indicated good biocompatibility with values higher than 70% (Figure 9H).

The neutral red analysis measures dye uptake and concentration within the lysosomes thus measuring staining capacity of live cells [109]. The fact that similar trends are not identified for this assay at all test sample concentrations, the cells can be considered to exhibit cytostatic effects at some point. This may mean that the introduction of the test compounds may have inhibited cell growth but does not necessarily facilitate cell obliteration or death. The observed cellular responses which occurred because of cell (Hep G2 and Caco2) exposure to different concentrations of the test samples can be described as dose-dependent and bi-phased cellular events which are related to the phenomenon referred to as, hormesis, which is a bimodal adaptive response of a typical biological systems (cells in this case) to external stressors (such as drugs/medicines, excipients, pH, temperature changes) [110,111]. In conclusion, the drug loaded suspension formulation developed showed no significant toxic effect on both model cells examined here. In other words, this pyrazinamide/rifampicin loaded formulation is potentially biocompatible.

## 4. Conclusions

This study details the systematic development of a novel multiparticulate reconstitutable suspension formulation containing fixed dose first line antitubercular agents (150 mg rifampicin/300 mg pyrazinamide per 5 mL) which can be potentially useful for the management of tuberculosis (both pulmonary and disseminated) in the paediatric population. The reconstitutable oral suspension contained the recommended levels of antitubercular agents according to the World health organization’s recently amended body-weight dosing requirements for minors and was successfully prepared by employing a combination of a solid–liquid dispersion, drying and mechanical milling. The production and optimization processes were facilitated by one-variable-at-a-time and high-performance Box–Behnken experimental design strategies. The optimized formulation was a free flowing microstructured powder with a reddish-brown appearance and desirable reconstitution and sedimentation properties in an aqueous medium. The production yield was high with negligible losses recorded and the formulation was biocompatible at the cellular level, environmentally stable (either in the dry or hydrated state), and displayed no destructive intrinsic drug-excipient interactions. Given the present global shortage of such preparations, particularly for the first line antitubercular medications, the result of this study serves as a significant scientific contribution to current global efforts towards improving pharmaceutical formulations for tuberculosis management in children and adolescents, closing the gaps created by the supply shortages of such needed dosage forms as well as achieving zero deaths within this age group (0–19 years). Besides, the drug delivery system developed here, either as is or modified, may also find use in the adult population suffering from active tuberculosis disease, particularly those having trouble in swallowing.

## Figures and Tables

**Table 1:** Levels of independent variables utilized for the synthesis of the reconstitutable suspensions.

Independent Variables	Levels/Limits		
	Lower (−1)	Middle (0)	Upper (+1)
Factor A *	0	1	2
Factor B **	3	4	5
Factor C ***	6	7	8

* Factor A: 0 = 8.00 mL WAT + 13.00 mL ETH + 0.00 mL GLY; 1 = 10.50 mL WAT + 15.50 mL ETH + 2.00 mL GLY; 2 = 13.00 mL WAT + 18.00 mL ETH + 4.00 mL GLY. ** Factor B: 3 = 0.15 g PRIM + 0.25 g PVA + 0.20 g PVP + 0.15 g MC + 0.10 g XY + 0.05 g PX + 0.10 g MS; 4 = 0.30 g PRIM + 0.45 g PVA + 0.60 g PVP + 0.50 g MC + 0.25 g XY + 0.30 g PX + 0.35 g MS; 5 = 0.60 g PRIM + 0.65 g PVA + 0.90 g PVP + 1.00 g MC + 0.50 g XY + 0.70 g PX + 0.50 g MS. *** Factor C: 6 = 50.00 mg KCl + 50.00 mg NaCl + 0.50 g STRW; 7 = 75.00 mg KCl + 75.00 mg NaCl + 0.75 g STRW; 8 = 100.00 mg KCl + 100.00 mg NaCl + 1.00 g STRW.

**Table 2:** Box–Behnken experimental design layout.

Reconstitutable Suspension	Factor A (X_1_)	Factor B (X_2_)	Factor C (X_3_)
RS 1 *	1	4	7
RS 2	2	4	6
RS 3	1	5	6
RS 4	0	3	7
RS 5 *	1	4	7
RS 6	1	3	6
RS 7	2	5	7
RS 8	2	4	8
RS 9	1	5	8
RS 10	0	5	7
RS 11 *	1	4	7
RS 12	0	4	8
RS 13	2	3	7
RS 14	1	3	8
RS 15	0	4	6

Note: * depicts the three experimental design center points. Each reconstitutable FDC suspension contained 150 mg rifampicin and 300 mg pyrazinamide per 5 mL of the reconstituted formulation.

**Table 3:** Set response parameter limits for predicting the optimized suspension formulation.

Response Parameters	Optimization Goal	Lower	Target	Upper	*R^2^* (%)	*p*-Value
Y_1_ * (%)	Target	68.700	80.000	94.300	84.800	0.000
Y_2_ * (degrees)	Minimum	11.300	15.700	24.300	89.460	0.019
Y_3_ * (strokes/second)	Target	1.675	1.800	1.990	78.790	0.001
Y_4_ *	Target	0.400	1.000	1.060	93.400	0.007

* Note: Y_1_ = Percentage yield, Y_2 =_ Angle of repose, Y_3_ = Average resuspension rate, and Y_4_ = Sedimentation indicator.

**Table 4:** Response parameters generated for the fifteen experimental design formulations.

Formulation	Y_1_ (%)	Y_2_ (°)	Y_3_ (Strokes/Seconds)	Y_4_
RS 1 *	93.30 ± 6.51	22.30 ± 0.58	1.69 ± 0.01	1.06 ± 0.05
RS 2	79.30 ± 7.51	21.00 ± 1.73	1.88 ± 0.19	0.78 ± 0.08
RS 3	83.30 ± 7.77	15.30 ± 3.21	1.73 ± 0.03	0.87 ± 0.13
RS 4	83.00 ± 11.27	12.70 ± 2.52	1.87 ± 0.02	0.76 ± 0.27
RS 5*	92.30 ± 3.51	24.00 ± 0.00	1.88 ± 0.01	1.06 ± 0.00
RS 6	75.30 ± 10.69	24.30 ± 1.53	1.80 ± 0.01	0.40 ± 0.05
RS 7	68.70 ± 3.51	15.70 ± 2.52	1.94 ± 0.08	0.92 ± 0.14
RS 8	76.30 ± 1.15	21.70 ± 1.15	1.96 ± 0.00	1.01 ± 0.02
RS 9	81.30 ± 4.93	11.30 ± 1.53	1.85 ± 0.05	0.99 ± 0.02
RS 10	88.00 ± 6.24	12.00 ± 1.00	1.71 ± 0.02	0.97 ± 0.00
RS 11 *	94.30 ± 2.52	23.00 ± 1.00	1.71 ± 0.01	1.05 ± 0.02
RS 12	79.30 ± 3.06	13.00 ± 0.00	1.84 ± 0.13	0.99 ± 0.02
RS 13	90.70 ± 4.16	21.00 ± 1.00	1.79 ± 0.06	0.48 ± 0.07
RS 14	76.70 ± 4.04	21.70 ± 2.52	1.99 ± 0.12	0.44 ± 0.04
RS 15	82.70 ± 8.14	12.00 ± 1.73	1.68 ± 0.03	0.74 ± 0.11

Note: * Experimental design center points. Y_1_—percentage yield; Y_2_ —angle of repose; Y_3_—average resuspension rate and Y_4_—sedimentation indicator.

**Table 5:** Optimized suspension formular and pointers showing experimental design reliability.

Optimized Formular (RS_opt_)	Indicators of Design Template Validity					
	Responses	Predicted	Experimental	*R^2^* (%)	*p*-Value	Desirability Level
Factor A = 0	Y_1_ (%)	91.562	96.000 ± 3.271	84.800	0.042	0.891
Factor B = 5	Y_2_ (°)	8.945	9.670 ± 1.150	89.461	0.037	1.000
Factor C = 8	Y_3_ (strokes/ seconds)	1.800	1.720 ± 0.011	93.400	0.016	1.000
	Y_4_	0.999	1.000 ± 0.010	78.792	0050	0.982
	Composite desirability level					0.967

**Table 6:** Representative mathematical models and their respective fit parameters.

Mathematical Model	Pyrazinamide		Rifampicin	
	*R^2^* Value	AIC	*R^2^* Value	AIC
Zero-order	0.99	48.77	0.99	40.75
First-order	0.84	85.05	−1.70	72.74
Second-order	0.43	94.92	−2.06	112.98
Higuchi	0.30	84.08	0.87	67.32
Korsmeyer-Peppas	0.96	61.52	−3.16	55.23
Michelis-Menten	0.90	90.01	−9.05	83.18

**Table 7:** Summary of analysis of variance results for quadratic model of particle size for reconstitutable powder formulation.

					95% Confidence Interval for Mean	
Samples	N	Mean (µm)	Standard Deviation	Standard Error	Upper Bound	Lower Bound
Placebo	1685.00	307.86	231.20	5.63	296.81	318.91
Drug-loaded	643.00	392.79	433.20	17.08	359.24	462.34
Pyrazinamide	339.00	205.59	108.60	5.90	193.98	217.20
Rifampicin	322.00	144.85	60.21	3.35	138.25	151.46

**Table 8:** Stability indicators for dry suspension powder over 8 weeks.

**Stability Indicators**		**Varying Storage Conditions over 8 Weeks**		
	**Week 0**	**Week 4**		
		I *	II *	III *
Yield (%)	93.33 ± 1.67	91.63 ± 3.13	90.06 ± 0.97	90.20 ± 4.45
Angle of repose (°)	12.37 ± 1.37	11.86 ± 0.80	12.69 ± 0.81	12.44 ± 0.63
Drug content (%)	**Rifampicin**			
	97.61 ± 0.02	97.01 ± 0.01	99.05 ± 0.02	97.21 ± 0.01
	**Pyrazinamide**			
	97.23 ± 2.57	98.12 ± 0.03	96.23 ± 0.01	98.03 ± 0.02
Colour change	4.00 ± 0.00	3.80 ± 0.45	2.40 ± 0.55	4.00 ± 0.00
Odour	5.00 ± 0.00	4.60 ± 0.55	2.80 ± 0.45	4.80 ± 0.45
**Stability indicators**		**Varying storage conditions over 8 weeks**		
	**Week 0**	**Week 8**		
		I *	II *	III *
Yield (%)	93.33 ± 1.67	90.13 ± 2.73	90.13 ± 2.73	88.27 ± 2.28
Angle of repose (°)	12.37 ± 1.37	12.26 ± 1.27	12.27 ± 1.07	12.27 ± 1.07
Drug content (%)	**Rifampicin**			
	97.61 ± 0.02	96.28 ± 0.01	97.11 ± 0.02	96.24 ± 0.00
	**Pyrazinamide**			
	97.23 ± 2.57	93.00 ± 0.01	93.02 ± 0.01	94.32 ± 0.02
Colour change	4.00 ± 0.00	3.60 ± 0.55	2.40 ± 0.55	3.60 ± 0.55
Odour	5.00 ± 0.00	4.40 ± 0.55	2.80 ± 0.45	4.60 ± 0.55

* Note: I—under room conditions (25 °C ± 2 °C/60 ± 5% RH); II—in a dark enclosure (25 °C ± 2 °C/60 ± 5% RH); III—refrigerated (5 °C ± 2 °C/35 ± 5 % RH).

**Table 9:** Measured indicators of stability for the reconstituted suspension.

**Stability Indicators**		**Varying Storage Conditions over 14 Days**		
	**Day 0**	**Day 7**		
		I *	II *	III *
Rate of redispersibility (stroke/seconds)	1.40 ± 0.02	1.80 ± 0.00	1.57 ± 0.00	1.76 ± 0.00
Sedimentation	2.30 ± 0.06	0.81 ± 0.02	0.91 ± 0.08	0.97 ± 0.02
Drug content (%)	**Rifampicin**			
	97.61 ± 0.02	97.00 ± 0.01	99.00 ± 0.02	97.22 ± 0.01
	**Pyrazinamide**			
	97.23 ± 2.57	98.00 ± 0.03	96.04 ± 0.01	98.34 ± 0.02
pH	6.95 ± 0.13	7.05 ± 0.01	7.79 ± 0.02	7.65 ± 0.04
Taste	5.00 ± 0.00	4.40 ± 0.55	4.40 ± 0.55	4.80 ± 0.45
Smell	5.00 ± 0.00	5.00 ± 0.00	4.40 ± 0.55	5.00 ± 0.00
Appearance	4.00 ± 0.00	3.80 ± 0.45	3.60 ± 0.55	3.80 ± 0.45
Colour	4.00 ± 0.00	3.40 ± 0.55	3.20 ± 0.45	3.60 ± 0.55
**Stability indicators**		**Varying storage conditions over 14 days**		
	**Day 0**	**Day 14**		
		I *	II *	III *
Rate of redispersibility (stroke/seconds)	1.40 ± 0.02	2.00 ± 0.00	2.00 ± 0.00	2.11 ± 0.19
Sedimentation	2.30 ± 0.06	0.7 ± 0.08	0.91 ± 0.08	0.86 ± 0.02
Drug content (%)	**Rifampicin**			
	97.61 ± 0.02	96.34 ± 0.01	97.00 ± 0.02	96.00 ± 0.00
	**Pyrazinamide**			
	97.23 ± 2.57	93.19 ± 0.01	93.00 ± 0.01	94.00 ± 0.02
pH	6.95 ± 0.13	8.29 ± 0.05	7.59 ± 0.05	8.16 ± 0.04
Taste	5.00 ± 0.00	4.40 ± 0.55	4.00 ± 0.00	4.60 ± 0.55
Smell	5.00 ± 0.00	4.00 ± 0.00	4.20 ± 0.45	4.60 ± 0.55
Appearance	4.00 ± 0.00	3.60 ± 0.55	3.00 ± 0.00	3.40 ± 0.55
Colour	4.00 ± 0.00	3.60 ± 0.50	3.00 ± 0.00	3.60 ± 0.55

* Note: I—under room conditions (25 °C ± 2 °C/60 ± 5% RH); II—in a dark enclosure (25 °C ± 2 °C/60 ± 5% RH); III—refrigerated (5 °C ± 2 °C/35 ± 5% RH).

## Data Availability

Not applicable.
